# The protective and chemotherapeutical role of amygdalin in induced mammary cancer in experimental mice and upregulation of related genes

**DOI:** 10.1038/s41598-025-93620-2

**Published:** 2025-03-17

**Authors:** Afaf D. Abdel Mageid, Ibrahim M. Abdel-Wadoud, Elsayed I. Salim, Thamer Aljutaily, Hassan Barakat, Huda Aljumayi, Khadija S. Radhi, Sami O. Almutairi, Tarek A. Ebeid

**Affiliations:** 1 Biochemistry and Molecular Biology, Faculty of Veterinary Medicine, Benha University, Moshtohor, 13736 Egypt; 2 Department of Zoology, Faculty of Science, Tanta University, Tanta, Egypt; 3 Department of Food Science and Human Nutrition, College of Agriculture and Food, Qassim University, 51452 Buraydah, Saudi Arabia; 4 Department of Food Science and Nutrition, College of Sciences, Taif University, P.O. Box 11099, 21944 Taif, Saudi Arabia; 5 Qassim Health Cluster, Ministry of Health, Riyadh, Saudi Arabia; 6 Department of Animal and Poultry Production, College of Agriculture and Food, Qassim University, 51452 Buraydah, Saudi Arabia

**Keywords:** Amygdalin, Tamoxifen, Protective, Cancer therapy, Mammary cancer, Caspase-3, BcL-2, P53, TNF-α, Health, Biochemistry, DNA, Breast cancer, Cancer therapy, Predictive markers, Nutrition therapy

## Abstract

Breast cancer is a prominent health issue among oncological diseases in emerging nations. The study sought to assess the significant function of amygdalin as a protective and chemotherapeutical substance in combating this lethal condition, either independently or in conjunction with tamoxifen therapy. Breast cancer in mice was induced by 7,12-Dimethylbenz(a)anthracene (DMBA). Mice were divided into six groups, 15 mice in each group. (i) control group, (ii) carcinogenic group, (iii) tamoxifen-treated group, (iv) Amygdalin-treated group, (v) (Amygdalin + tamoxifen) group, (vi) Amygdalin protective group. Results revealed that DMBA-induced breast cancer caused a significant increase in biochemical parameters such as CEA, CA15.3, CA125, PRL, E2, urea, creatinine, ALT, AST, and ALP and a substantial increase in gene expression of TNF-α and BcL-2. In contrast, amygdalin administrations alone or in co-administration with tamoxifen could ameliorate breast cancer by declining TNF-α, BcL-2 and attenuating the biochemical parameters. Amygdalin administrations showed a significant increase in SOD and GPx antioxidants and upregulation of Caspase-3 and P53 in breast tissue. Moreover, flow cytometric analysis revealed that amygdalin administrations were correlated with CD20 and CD44 and promoted the cell cycle and apoptosis in carcinogenic mice. Indeed, the above results were confirmed by the histopathological examinations, which showed that the DMBA group had proliferated microductular carcinoma with marked mononuclear inflammatory cell infiltration, which decreased by the Amygdalin administrations. In conclusion, amygdalin administration may be effective in preventing breast cancer and exhibiting chemotherapeutic properties.

## Introduction

Breast cancer is a subtype that develops in the breast tissue itself, typically in the ductal carcinoma in situ (DCIS) or lobules that feed the ducts. Sometimes, DCIS develops into an aggressive malignancy, while in others, the cells remain in the ducts and never act deeper or spread to the lymph nodes or other organs^1^. Different from ductal carcinoma in situ, lobular carcinoma in situ is the cause of the second subtype of breast cancer (LCIS). In LCIS, cancer cells grow in milk-producing glands but do not invade the lobular wall. Lobular neoplasia is another name for LCIS. Noninvasive breast cancers include this and ductal carcinoma in situ (DCIS). However, unlike DCIS, it does not appear to progress into an aggressive malignancy if treatment is delayed^2^. Various chemicals have been known as etiological factors in human carcinogenesis. Most of these chemicals are genotoxic in different short-expression assays. Most are metabolically activated to the reactive middle that binds directly to DNA, perhaps leading to genetic damage that results in cancer. Examples of these chemicals are 7,12-dimethyl benz(a)anthrathene^3^. When cancer has spread to other parts of the body (metastatic), various treatments such as chemotherapy, hormonal therapy, or radiation may be used to reduce symptoms, improve quality of life, and extend survival^4^. Thus, tamoxifen, a hormonal treatment, prevents breast cancer by modulating the selective estrogen receptor (SERM)^5^.

Amygdalin was first isolated in 1830 by two French chemists Robiquet and Boutron-Charlard. They discovered amygdalin (which was named “emulsin”) from bitter almonds in 1837^6^. Orally administered amygdalin is thought to be hydrolyzed into prunasin and glucose by human digestive enzymes, and prunasin is further degraded into mandelonitrile in the small intestine. The transformation of mandelonitrile into benzaldehyde and cyanide and the subsequent toxicity are mainly due to gut microflora^7^. Evidence shows that amygdalin can fight free radicals and reduce inflammation^8^. It has anti-inflammatory, antimicrobial, antioxidant, and immunomodulatory properties^9–11^. Lea et al.^12^ stated in the Journal of the National Cancer Institute (JNCI) that amygdalin inhibits tumor growth. Hydrocyanic acid, which has anti-cancer effects, and benzaldehyde, which has analgesic properties, are the products of amygdalin decomposition. As a result, it can be used to combat cancer and alleviate the pain^13^. Amygdalin’s anti-tumor effect has been a crucial topic in recent years. Amygdalin’s anti-cancer properties are governed by carcinogenic chemicals broken down in the body and kill tumor cells, cutting off cancerous cells’ access to nutrition and halting their growth. It has been processed and used to treat malignancies using conventional medicine in 20 countries, including the US, Italy, Japan, and China^14^. Amygdalin’s benefits have been studied for decades, and the findings have been consistent across various medical illnesses like leprosy, colorectal cancer, asthma, bronchitis, and others^15,16^. Its molecular structure includes the analgesic component benzaldehyde^13^. However, more studies are needed to determine how effective its anti-cancer activity is. Hydrocyanic acid emission during enzymatic hydrolysis may be responsible for this capacity^17^. Amygdalin’s toxicity to healthy cells and poor pharmacokinetic qualities have raised doubts about its usefulness as an anti-cancer medication^18^. It is hypothesized that the primary anti-cancer activity is reduced cancer cell proliferation caused by a lack of carcinogenic chemicals. The incidence of many cancers can be reduced by a process known as apoptosis^19^, which involves cutting off cancer cells’ access to nutrients^20^. More efforts have been struggled to realize natural and synthetic anti-tumors with antioxidant efficacy. Therefore, some trials have been conducted, and the results showed a dual synergistic effect between synthetic anti-tumor and natural agent^21^.

Throughout the ages, people have turned to natural medicines to combat illness. Amygdalin, commonly known as bitter apricot, laetrile, and almond, is a cyanogenic molecule in the aromatic cyanogenic glycoside class; its chemical formula is D-mandelonitrile-D-glucoside-6-glucoside. Amygdalin can be found in the seeds of many different plants, although it is most abundant in rosaceous plants like apricots, peaches, cherries, plums, etc.^22,23^. Amygdalin is a vital active medicinal ingredient found in almonds and the seeds of some plants of the Rosaceae family^24,25^. It’s a naturally occurring biomolecule that can be discovered in the seeds of many different plants^9^. Therefore, this investigation aimed to examine the anti-tumor effects of the amygdalin molecule against mammalian tissue cancer in mice to examine the in vivo anti-tumor effects of the amygdalin molecule against mammalian tissue cancer in mice and explore the dual synergistic effect between synthetic anti-tumor of tamoxifen and amygdalin which not yet proven.

## Materials and Methods

### Chemicals

#### 12-Dimethylbenz[a]anthracene

The most commonly utilized chemical carcinogen is DMBA with chemical formula C20H16, molecular weight 256.341 g mol^-1^, in powder and dissolved in 100% sesame oil, purchased from Sigma-Aldrich Chemical Co., ID. NO. 24891046, provided by the Egyptian International Center for Import Cairo, Egypt); it is used at an oral dose level (50 mg kg^-1^ once a week) for 4 weeks^26^.

#### Tamoxifen (Nolvadex-D) ®

A chemical anti-cancer drug manufactured by (AstraZenica group. AstraZenica©2000, UK limited) in the form of a tablet, and each tablet contains (20 mg). It is dissolved in distilled water with the chemical formula C26H29NO and a molecular weight of 371.515 g mol^-1^. Tamoxifen was administered orally at a dose (50 mg kg^-1^ b.wt/day) for 4 weeks^27^.

#### Amygdalin

D-Amygdalin was purchased from Sigma-Aldrich Chemical Co. (St. Louis, MO, USA); ID.NO. 24891046; in the form of crystalline powder with chemical formula C20H27NO11, molecular weight 457.44 Dalton. Amygdaline was dissolved in 100% corn oil and administered orally at a dose of (o.6 mg kg^-1^ b. wt/day)^28^.

### Experimental animals

Ninety female albino mice, 4 to 6 weeks of age and weighing 18 to 35 g, were used in this study^29^. Mice were purchased from the Animal Research Center, Faculty of Veterinary Medicine, Benha University. Animals were isolated in metal cages with controlled temperatures and diets for the duration of the study. The animals were fed a steady diet and had access to water *ad libdium*. Mice were allowed to acclimatize at the animal facility for at least one week before the start of the experiment. The Ethical Committee of the School of Veterinary Medicine at Benha University reviewed and approved all experimental procedures and provided ethical approval No. BUFVTM on 18–01-23. All research was also performed following relevant guidelines and regulations.

### Experimental design

Female mice were randomly split into five groups, fifteen in each, housed in separate cages and treated as Group I (Normal control): untreated female mice served as controls for all experimental groups. Group II (carcinogenic induced group): female mice administrated oral DMBA at a dose of (50 mg kg^-1^ b.wt orally once a week) in sesame oil at 5 weeks of age for 4 weeks^26^. Group III: (Tamoxifen treated group) Female carcinogenic mice received tamoxifen orally at a dose (50 mg kg^-1^ b.wt/day) dissolved in distilled water for 4 weeks^27^. Group IV: (Amygdalin treated group) female mice administrated with amygdalin (0.6 mg kg^-1^ b.wt/day) orally in 100% corn oil for 4 weeks^28^ Group V: (Tamoxifen + Amygdalin treated group) female carcinogenic mice treated orally with tamoxifen (50 mg kg^-1^ b.wt/day) dissolved in distilled water^27^ and amygdalin (0.6 mg kg^-1^ b.wt/day) in 100% corn oil for 4 weeks^28^. Group (vi): (Amygdalin protection group), female mice administrated with amygdalin (0.6 mg kg^-1^ bw/day) dissolved in 100% corn oil orally for 4 weeks^28^ then administrated with DMBA (50 mg kg^-1^ b.wt. orally once a week) in sesame oil at 5 weeks age for 4 weeks^26^ along with amygdalin as above. During the whole experiment, the ARRIVE guidelines have been followed.

### Sampling

#### Blood Sampling

Blood samples were taken from mice at the end of the experiment and divided into two parts: one was put in a plain tube to obtain the serum after centrifugation at 3000 rpm for 15 min. The clear serum was collected using an automated pipette, placed in a dry sterile samples tube, and stored in the freezer at -20 °C till carrying the biochemical analysis for tumor markers (carcinoembryonic antigen (CEA), cancer antigen 15.3 (CA15.3) and cancer antigen 125 (CA125)); steroid hormones (prolactin (PRL) and estrogen (E2)); hepatic enzymes such as alanine transaminase (ALT), aspartate transaminase (AST) and alkaline phosphatase (ALP); and renal functions such as urea and creatinine.

#### Tissue samples (breast tissues):

According to Marquardt et al.^30^, mice were anesthetized using intramuscular injection of ketamine/xylazine hydrochloride mix (80/10 v:v) before being sacrificed when their experimentation time was up, blood samples collected, and then cervically dislocated. The breast tissues were extirpated and cut into 4 parts. 1st part: oxidative stress biomarkers analyses (superoxide dismutase (SOD), and glutathione peroxidase (GPx); 2nd part: gene expression (Caspase-3, Bcl2, TNF-α, and P53); 3rd part: flowcytometric analysis for (CD20, CD44, cell cycle, and apoptosis); and 4th part: the tissue was fixed in 10% neutral buffered formalin till processing histopathological examination. All specimens were kept at—80°C till analysis, except the 4th part.

### Assay Methods

#### Serum biochemical parameters

ALT and AST were carried according to methods described by Young^31^, ALP^32^, urea^33^, and creatinine ^34^ using JENWAY 6051 Colorimeter U.K device with their specific Spectrum GmbH Company kits (CAT. NO. 264001; 260001; 216001; 318001; 235001, respectively). CEA, CA15.3, CA125, E2, and PRL concentrations were determined using a Chinese instrument, the AutoLumo A1000, and the manufacturer-recommended reagent kits (Autobio Diagnostics Co., LTD), CAT. NO. CL0205-2, CL0210-2, CL0209-2, CL1105-2, and CL1103-2, respectively, as described by Sugarbaker^35^, Geraghty et al.^36^, Haga et al.^37^, Martin and Rotten^38^, and Uotila et al.^39^, respectively.

#### Tissue oxidative stress biomarkers

Tissues from the mammary gland were collected and washed in phosphate-buffered saline (PBS) containing 0.16 mg ml^-1^ heparin to eliminate debris such as blood cells and clots. Using a cleaner, a gram of breast tissue was homogenized in 5 mL of cold buffer (i.e., 50 mM potassium phosphate, PH 7.5 1 mM EDTA). Aliquots of tissue homogenates were centrifuged by a cooling centrifuge at 4000rpm for 20 min. The supernatant was removed and stored at -20°C till the oxidative stress biomarkers (SOD and GPx) were determined by Spectro nanodrop with commercial kits Biodiagnostic, Cairo, Egypt; CAT. NO. SD2521 and GP2524, respectively, according to Nishikimi et al.^40^ and Paglia and Valentine^41^, using the supernatants from the homogenates centrifuged at 8000 rpm for 20 min at 4 °C.

#### Gene expressions of caspase-3, BcL-2, P53, and TNF-α

Following the manufacturer’s instructions, total RNA was isolated from the frozen breast tissue using the RNeasy® Mini kit (Qiagen)^42^. Quantitative and qualitative RNA assessments were made using spectrophotometry (SPECTRO star Nano, BMG Labtech Co., Ortenberg, Germany). High-capacity cDNA Reverse Transcription Kits were used to convert 1000 ng of total RNA into single-stranded cDNA following the manufacturer’s instructions (Applied Biosystems). Gene analyses were performed with real-time time-PCR using sense and anti-sense primers (Table 1)^43^. PCR reactions for each gene were carried out for each analyzed sample. Each PCR reaction consisted of 1.5 µl of 1µg µl^-1^ cDNA, 10 µl SYBR Green PCR Master Mix (QuantiTect SYBR Green PCR kit, Qiagen), 1µM of each forward and reverse primer for tested genes while 1µM of forward and 1.5 µM reverse primer for (housekeeping) gene (β-actin) and nuclease-free water to a final volume of 20 µl. Reactions were then analyzed on an applied Biosystem 7500 Fast Real time PCR Detection system under the following conditions: 95°C for 10 min (holding stage) and 40 cycles of 95°C for 15 s (denaturation stage) followed by 60°C for 1 min (annealing and extension stage). Changes in gene expression were calculated from the obtained cycle threshold (Ct) values provided by real-time PCR instrumentation using the comparative CT method to a reference (housekeeping) gene (β-actin)^44^.

**Table 1:** Gene sense and anti-sense primers for real time-PCR.

Gene name	Primer sequence
TNF-α	F: AAATGGGCTCCCTCTCATCAGTTC R: TCCGCTTGGTGGTTTGCTACGAC
BcL-2	F: TATATGGCCCCAGCATGCGA R: GGGCAGGTTTGTCGACCTCA
Caspase-3	F: GTGGAACTGACGATGATATGGC R: CGCAAAGTGACTGGATGAACC
P53	F: CTACTAAGGTCGTGAGACGCTGCC R: TCAGCATACAGGTTTCCTTCCACC
β-actin	F: ACCCCAAAGCCAACAGA R: TCTCAGCTGTGGTGTGAAG

### Tissue flowcytometric analysis for CD44, CD20, cell cycle, apoptosis

Before the flowcytometric analysis, fresh tissue specimens were preserved in isotonic saline and consequently prepared. The tissue specimens were washed with isotone Tris EDTA buffer, 3.029 gm of 0.1 M Tris (hydroxymethylaminomethane, 1.022 gm of 0.07 M sodium chloride (ADWIC), and 0.47 gm of 0.005 M EDTA then, dissolved in 250 ml of distilled water and adjusted the pH to 7.5 by using 1 N HCl. Cell suspension was centrifuged at 1800 rpm for 10 min, where the supernatant was aspirated. After centrifugation and aspiration of the supernatant, the cells were fixed in ice-cold 96–100% ethanol in approximately (1 ml for each sample). Flow cytometry was used to examine the expression of CD20 and CD44 in breast tissue using fluorescently labeled mouse anti-human antibodies (BD PharmingenTM, PE mouse anti-human CD20, and PE mouse anti-human CD44, respectively), as well as the cell cycle using Propidium Iodide (Sigma Aldrich, St. Louis, USA) and Annexin V (BD PharmingenTM, FITC Apoptosis Kit, Cat no 556547, BD Biosciences)^45^. The computer program for mathematical analysis was modified to examine cell distribution histograms and estimate cells at different stages of the cell cycle^46^.

### Histopathological examination

The mice were slaughtered, their breasts removed, and the tissue was immediately collected in a 10% formalin solution and treated using the paraffin procedure. Paraffin beeswax tissue blocks were prepared for cutting at 4 µm by sliding microtome. After microtome sectioning, the tissue sections were deparaffinized and immediately stained with hematoxylin–eosin (H&E). The stained sections were diagnosed for histopathological alterations in breast architecture, and their photomicrographs were taken according to Banchroft and Gamble^47^. Subsequently, the results of undefined experimental groups were re-diagnosed by two pathologists to confirm the observation of the result.

### Statistical analysis

The statistical package for social science (SPSS, 22.0 software) was performed according to Steel et al.^48^, and values of *p* < 0.05 were considered significant. The results were analyzed statistically using one-way analysis of variance (ANOVA), then Tukey’s multiple test was applied, and data were presented as mean ± SE.

## Results

### Effect on hepatic enzymes and renal functions

Results revealed that DMBA-induced breast cancer caused a significant increase in hepatic enzymes, in which AST was increased by 57% and ALP by 29% compared to the control group. Still, Amygdalin administration as a treatment improved hepatic enzymes by AST, which improved by 27%, ALT enhanced by 15%, and ALP improved by 5% compared to the DMBA group. They revealed a significant increase in renal functions, wherein urea was increased by 41% and creatinine by 61% compared to the control, while Amygdalin administration as a protection improved renal functions by urea, which decreased by 11% and creatinine decreased by 31% in compared to DMBA group., Table 2.

**Table 2:** Effect of Amygdalin administrations compared with tamoxifen therapy and DMBA carcinogen on hepatic and renal functions in DMBA-induced tumor mice.

Groups	AST U L^-1^	ALT U L^-1^	ALP U L^-1^	Urea mg dl^-1^	Creatinine mg dl^-1^
Control	35.00 ± 0.58^d^	19.00 ± 1.15^bc^	27.43 ± 0.97^d^	39.52 ± 1.26^c^	0.93 ± 0.03^d^
DMBA	55.67 ± 2.03^a^	19.00 ± 1.15^bc^	35.87 ± 2.17^b^	45.71 ± 0.83^a^	1.53 ± 0.09^a^
Tamoxifen Therapy	35.00 ± 0.58^d^	17.33 ± 0.88^bc^	29.33 ± 1.24^cd^	42.80 ± 0.00^b^	1.20 ± 0.00^b^
Amygdalin Treatment	40.00 ± 0.58^c^	16.33 ± 0.33^c^	33.43 ± 0.72^bc^	41.87 ± 0.93^bc^	1.10 ± 0.06^bc^
Tamoxifen + Amygdalin	55.33 ± 2.03^a^	20.00 ± 0.58^b^	34.90 ± 1.33^b^	40.71 ± 0.41^bc^	1.07 ± 0.03^bcd^
Amygdalin Protection	49.67 ± 1.45^b^	24.00 ± 0.58^a^	43.00 ± 1.91^a^	40.47 ± 0.47^bc^	1.03 ± 0.03^cd^

^a,b,c,d^: means in the same column with different subscripts are statistically different at (P˃0.05).

### Effect on steroid hormones

The results revealed that DMBA-induced breast cancer showed a significant increase in steroid hormones, in which PRL was increased by 41% and E2 by 193% compared to the control group, but Amygdalin in co-administration with tamoxifen improved steroid hormones by PRL which decreased by 13% compared to DMBA group, as well as E2 decreased by 15% with Amygdalin as a protective administration in compared to DMBA group, Table 3.

**Table 3:** Effect of Amygdalin administrations compared with tamoxifen therapy and DMBA carcinogen on steroid hormones in DMBA-induced tumor mice.

Groups	Estrogen (E2) Pg ml^-1^	Prolactin (PRL) ng ml^-1^
Control	30.90 ± 1.50^bc^	0.87 ± 0.01^b^
DMBA Carcinogen	88.46 ± 5.14^a^	1.23 ± 0.09^a^
Tamoxifen Therapy	34.07 ± 0.29^bc^	0.87 ± 0.01^b^
Amygdalin Treatment	35.01 ± 0.25^b^	0.90 ± 0.01^b^
Tamoxifen + Amygdalin	40.71 ± 0.41^bc^	1.07 ± 0.03^bcd^
Amygdalin Protection	32.03 ± 0.76^bc^	0.89 ± 0.01^b^

^a,b,c,d^: means in the same column with different subscripts are statistically different at (P˃0.05).

### Effect on tumor markers

The results revealed that DMBA-induced breast cancer showed a significant increase in tumor markers, in which CEA was increased by 201%, CA15.3 by 164%, and CA125 by 109% compared to the control group. On the contrary, Amygdalin in co-administration with tamoxifen declined the tumor markers by CEA, which decreased by 63%, CA15.3 by 60%, and CA125 by 46% compared to the DMBA group, Table 4.

**Table 4:** Effect of Amygdalin administrations compared with tamoxifen therapy and DMBA carcinogen on tumor markers in DMBA-induced tumor mice.

Groups	CEA ng ml^-1^	CA15.3 U L^-1^	CA125 U L^-1^
Control	0.54 ± 0.03^c^	0.70 ± 0.02^b^	2.16 ± 0.08^b^
DMBA	1.63 ± 0.05^a^	1.85 ± 0.07^a^	4.53 ± 0.18^a^
Tamoxifen Therapy	0.61 ± 0.04^bc^	0.71 ± 0.02^b^	2.30 ± 0.06^b^
Amygdalin Treatment	0.68 ± 0.03^b^	0.78 ± 0.04^b^	2.48 ± 0.10^b^
Tamoxifen + Amygdalin	0.59 ± 0.03^bc^	0.74 ± 0.05^b^	2.28 ± 0.10^b^
Amygdalin Protection	0.68 ± 0.02^b^	0.77 ± 0.03^b^	2.48 ± 0.08^b^

^a,b,c^: means in the same column with different subscripts are statistically different at (P˃0.05).

### Effect on Oxidative Stress

Breast tissueSOD antioxidant was increased by 2.5%, and breast tissue GPx antioxidant was increased in percentage of 123% with the said dose of Amygdalin protection administration when compared with the DMBA group. Still, SOD was downed by given DMBA by 2.9% and GPx by 48% compared to the control group, Table 5.

**Table 5:** Effect of Amygdalin administrations compared with tamoxifen therapy and DMBA carcinogen on oxidative stress in DMBA-induced tumor mice.

Groups	SOD U g^-1^	GPx U g^-1^
Control	1368.96 ± 4.71^a^	4675.30 ± 2 42.02^ab^
DMBA	1327.12 ± 17.97^b^	2388.34 ± 653.97^c^
Tamoxifen Therapy	1374.37 ± 0.22^a^	3987.94 ± 112.31^b^
Amygdalin Treatment	1375.12 ± 1.52^a^	4513.18 ± 404.33^ab^
Tamoxifen + Amygdalin	1368.37 ± 5.41^a^	4201.93 ± 303.25^ab^
Amygdalin Protection	1361.62 ± 1.95^a^	5349.68 ± 202.16^a^

^a,b,c^: means in the same column with different subscripts are statistically different at (P˃0.05).

### Effect on Gene Expressions of TNF-α, BcL-2, Caspase-3, and P53

TNF-α of breast tissue was significantly upregulated by DMBA when compared with the control group (from the value of control 1.05 ± 0.02 to the value 2.73 ± 0.04 of DMBA) and grossly downregulated by Amygdalin administration—asa protective substance (from the value 2.73 ± 0.04 of DMBA to the value 0.03 ± 0.01 of Amygdalin protective substance) along with BcL-2 of breast tissue was significantly upregulated by DMBA when compared with the control group (from the value of control 1.06 ± 0.02 to the value 1.54 ± 0.11 of DMBA) and grossly downregulated by Amygdalin– in coadministration with tamoxifen- with cancer induced by DMBA (from the value 1.54 ± 0.11 of DMBA to the value 0.62 ± 0.19 of Amygdalin with tamoxifen); however, Caspase-3 of breast tissue was significantly upregulated bycoadministration of Amygdalin with tamoxifen (from the value 0.55 ± 0.31 of DMBA to the value 2.42 ± 0.14 of Amygdalin with tamoxifen), as well as P53 of breast tissue wassignificantly upregulated by Amygdalin treatment (from the value 0.04 ± 0.02 of DMBA to the value 1.54 ± 0.14 of Amygdalin treatment) and both of them downed by given DMBA., Table 6.

**Table 6:** Effect of Amygdalin administrations compared with tamoxifen therapy and DMBA carcinogen on gene expressions in DMBA-induced tumor mice.

Groups	TNF-α	BCL-2	Caspase-3	P53
Control	1.05 ± 0.02^b^	1.06 ± 0.02^b^	1.15 ± 0.03^bc^	1.12 ± 0.02^b^
DMBA	2.73 ± 0.04^a^	1.54 ± 0.11^a^	0.55 ± 0.31^c^	0.04 ± 0.02^c^
Tamoxifen Therapy	0.06 ± 0.00^d^	1.27 ± 0.08^ab^	2.40 ± 0.05^a^	1.36 ± 0.06^a^
Amygdalin Treatment	0.70 ± 0.16^c^	0.99 ± 0.06^b^	1.68 ± 0.31^b^	1.54 ± 0.14^a^
Tamoxifen + Amygdalin	0.18 ± 0.07^d^	0.62 ± 0.19^c^	2.42 ± 0.14^a^	1.11 ± 0.03^b^
Amygdalin Protection	0.03 ± 0.01^d^	1.19 ± 0.07^b^	1.10 ± 0.15^bc^	1.06 ± 0.06^b^

^a,b,c,d^: means in the same column with different subscripts are statistically different at (P˃0.05).

### Effect on CD20, CD44, cell cycle, and annexin V by flowcytometry

**Fig. 1:**
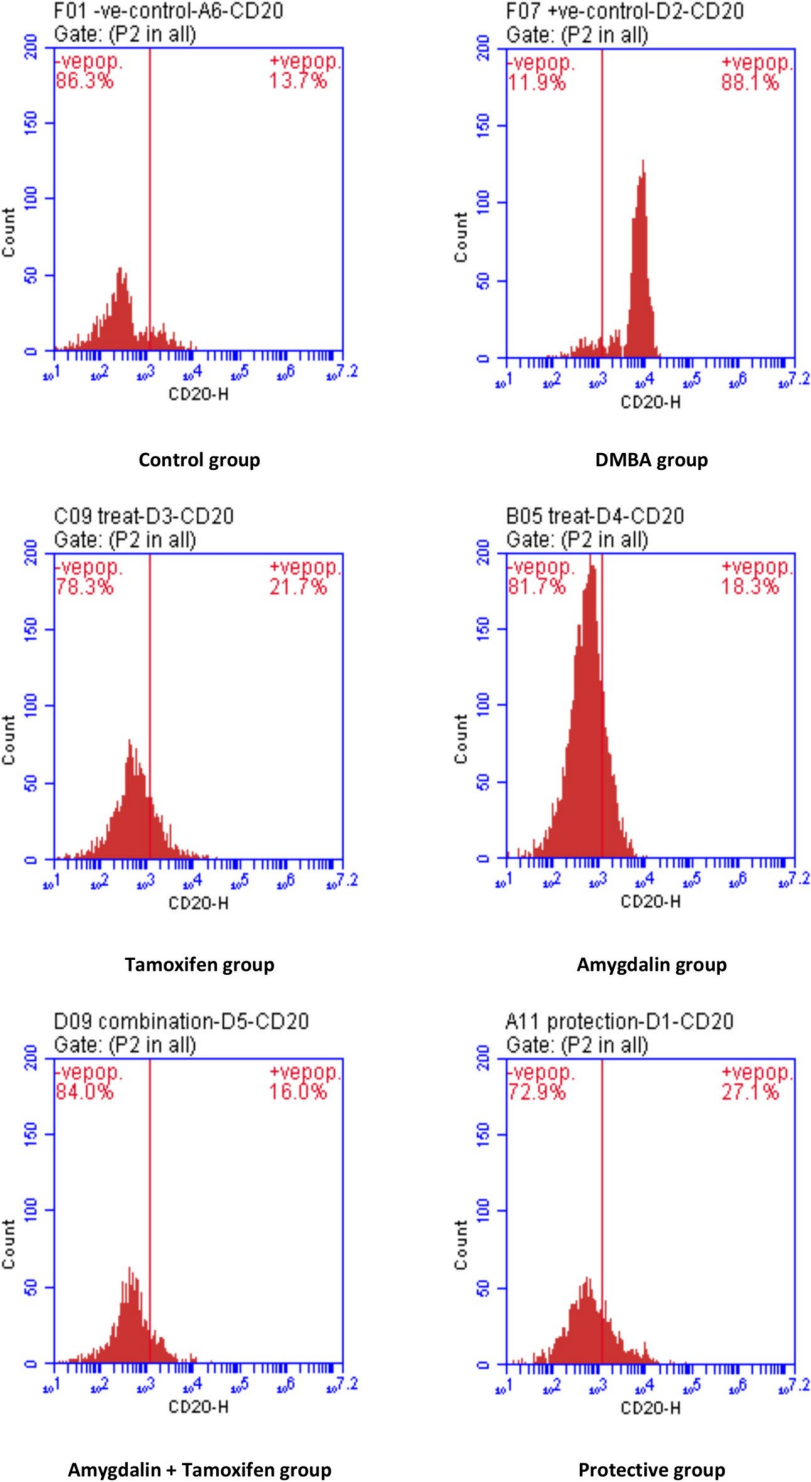
Flow cytometry charts of studied groups’ mammary (CD20) activities.

**Fig. 2:**
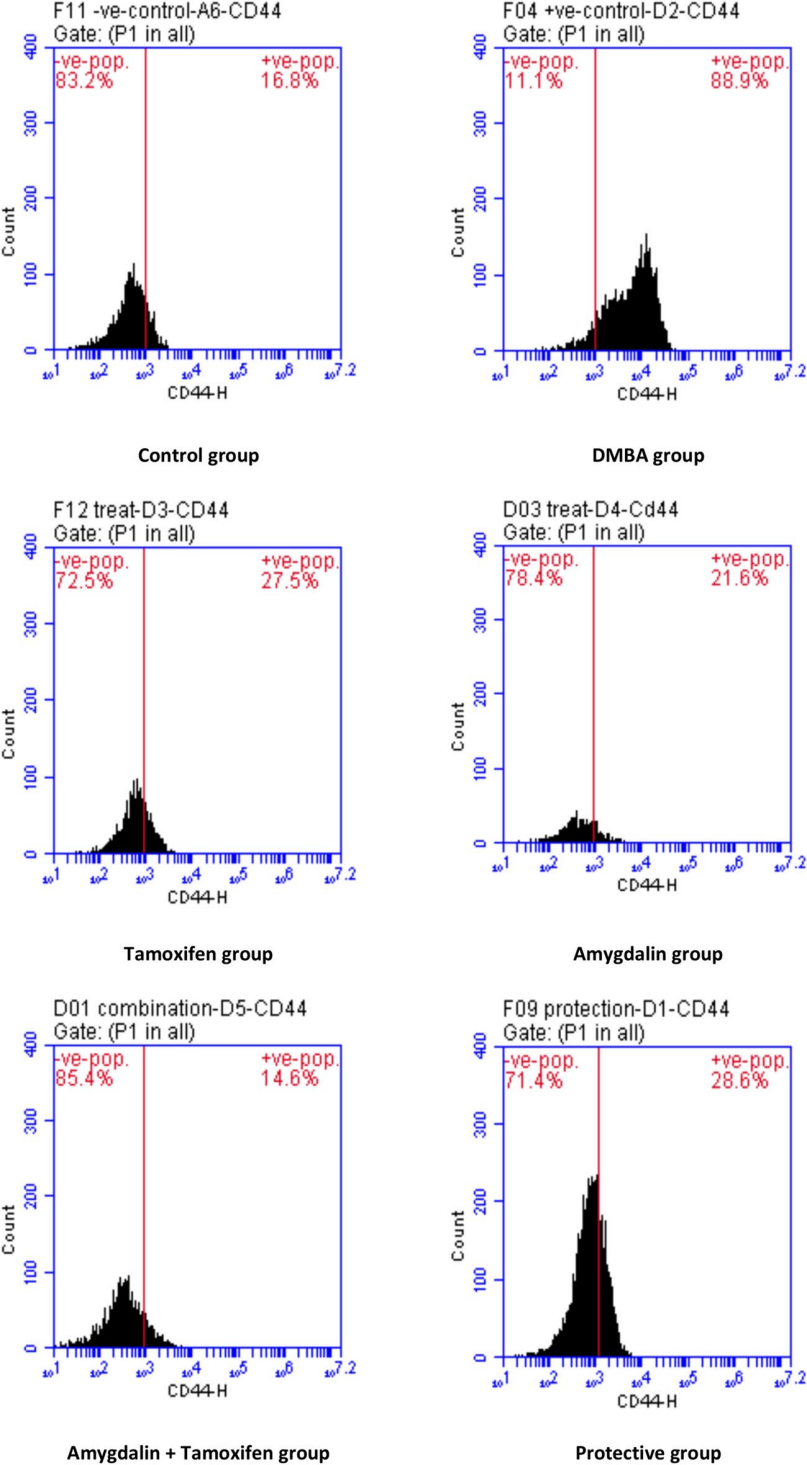
The flow cytometric histogram shows CD44 staining for different groups.

The results of CD20 and CD44 contents revealed significant increases in the breast tissue of DMBA mice when compared with the regular control group, while Amygdalin administrations—as a treatment, a protective substance, or co-administrated with tamoxifen therapy—exposed significant decrease when compared with DMBA carcinogenic group, as shown in Table 7 and Figs. 1 and 2.

**Table 7:** Effect of Amygdalin administrations compared with tamoxifen therapy and DMBA carcinogen on CD20 and CD44 in DMBA-induced tumor mice.

Groups	CD20	CD44
Control	11.60 ± 1.34 ^e^	14.07 ± 1.46 ^c^
DMBA	87.53 ± 1.16 ^a^	83.17 ± 5.39 ^a^
Tamoxifen Therapy	27.10 ± 3.41 ^bc^	27.10 ± 1.69 ^b^
Amygdalin Treatment	22.10 ± 2.99 ^cd^	22.70 ± 1.20 ^b^
Tamoxifen + Amygdalin	18.23 ± 1.24 ^de^	14.60 ± 0.35 ^c^
Amygdalin Protection	29.90 ± 2.65 ^b^	29.83 ± 0.79 ^b^

Data are presented as mean ± SE.

SE Standard Error.

^a,b,c,d,e^means in the same column with different subscripts are statistically different at (P˃0.05).

Furthermore, significant deregulation of cell distribution was observed in sub G1 phase (DNA fragmentation), G0/1 (cell viable), S (synthesis of DNA), and G2/M (arrest in the cell cycle) phases in cancer tissue of carcinogenic mice compared to those of the control mice. Pre-treatment with amygdalin ameliorated the deregulation of the cell population significantly; however, a pronounced increase in cell population was detected in the G1 phase and G0/1 phase. Otherwise, a significant decrease in cell population was seen in the S phase and G2/M phase compared to DMBA mice. Regarding the effect of treatments, amygdalin as a single treatment resulted in a cell population in the sub-G1 phase. Meanwhile, significant change was recorded in other phases compared to the control group.

**Fig. 3:**
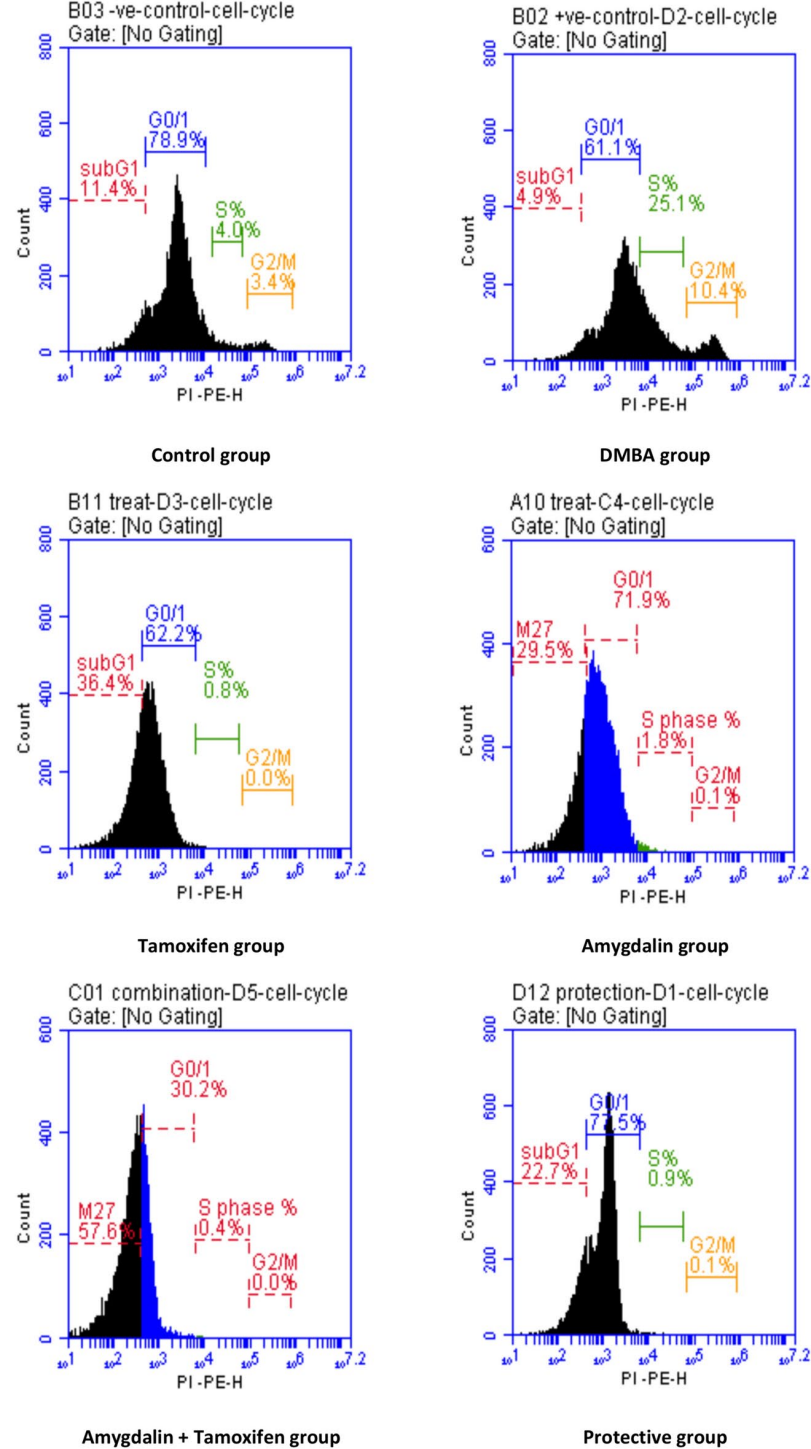
Flow cytometric analysis of cell cycle phases for different groups.

Furthermore, Amygdalin co-administration with tamoxifen therapy caused remarkable improvement in cell population distribution in the different cell cycle phases and recorded significant differences concerning DMBA mice. On the other hand, more remarkable cell arrest has occurred at the sub-G1 phase, S phase, and G2/M phase. However, an insignificant change in cell population was observed in the G1 phase compared to amygdalin as sole treatment, as shown in Table 8 and Fig. 3.

**Table 8:** Effect of Amygdalin administrations compared with tamoxifen therapy and DMBA carcinogen on cell cycle in DMBA-induced tumor mice.

Groups	Sub-G1	G0/1	S phase	G2 M
Control	10.33 ± 0.67 ^d^	79.47 ± 2.13 ^a^	6.03 ± 1.93 ^b^	3.50 ± 0.78 ^b^
DMBA	5.47 ± 0.38 ^e^	66.33 ± 2.64 ^ab^	20.47 ± 2.35 ^a^	10.43 ± 1.88 ^a^
Tamoxifen Therapy	31.60 ± 3.10 ^b^	64.77 ± 2.62 ^b^	1.50 ± 0.70 ^bc^	0.07 ± 0.07 ^c^
Amygdalin Treatment	32.00 ± 1.28 ^b^	64.20 ± 7.85 ^b^	1.87 ± 0.12 ^bc^	0.37 ± 0.22 ^c^
Tamoxifen + Amygdalin	60.40 ± 1.40 ^a^	30.53 ± 0.33 ^c^	0.43 ± 0.03 ^c^	0.00 ± 0.00 ^c^
Amygdalin Protection	21.97 ± 0.94 ^c^	71.87 ± 5.63 ^ab^	3.37 ± 2.42 ^bc^	0.87 ± 0.77 ^bc^

Data are presented as mean ± SE.

SE: Standard Error.

^a,b,c,d,e^: means in the same column with different subscripts are statistically different at (P˃0.05).

**Fig. 4:**
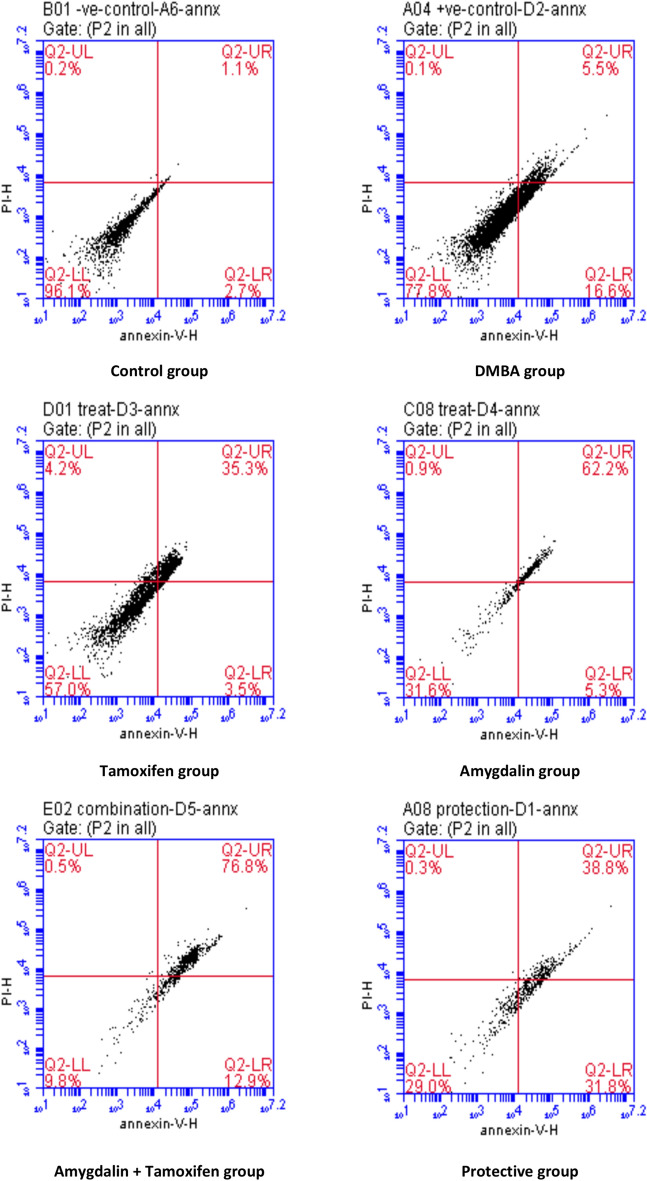
The flow cytometric analysis of the annexin-v kit for different groups is shown as a quadrant analysis.

Moreover, the results of Annexin V (LL) showed a significant increase in the breast tissue of DMBA mice compared with control mice and a significant decrease with Amygdalin administrations—as a treatment, a protective substance, or co-administrated with tamoxifen therapy—when compared with DMBA mice. Annexin V (UL, LR, and UR) results showed a significant decrease in the breast tissue of DMBA mice but a significant increase with Amygdalin administrations. On the other hand, tamoxifen therapy resulted in a considerable reduction in Annexin V (LL) and a substantial increase in Annexin V (UL, LR, and UR), as shown in Table 9 and Fig. 4.

**Table 9:** Effect of Amygdalin administrations compared with tamoxifen therapy and DMBA carcinogen on Annexin V in DMBA-induced tumor mice.

Group	UL	UR	LL	LR
Control	0.20±0.00^b^	1.47±0.63^d^	95.47±1.23^a^	2.93±0.56^c^
DMBA	0.10±0.00^b^	5.33±0.09^d^	79.47±1.08^b^	15.80±0.44^b^
Tamoxifen Therapy	3.03±1.22^a^	36.83±1.11^c^	54.33±2.19^c^	5.80±2.30^c^
Amygdalin Treatment	1.00±0.06^b^	60.40±2.48^b^	33.87±2.37^d^	4.70±0.35^c^
Tamoxifen+ Amygdalin	0.43±0.07^b^	72.60±2.48^a^	12.83±1.76^e^	14.17±0.78^b^
Amygdalin Protection	0.30±0.06^b^	37.97±2.66^c^	32.53±4.15^d^	29.20±1.66^a^

Data are presented as mean ± SE.

SE: Standard Error.

^a,b,c,d,e^: means in the same column with different subscripts are statistically different at (P˃0.05).

### Histopathological findings

**Fig. 5:**
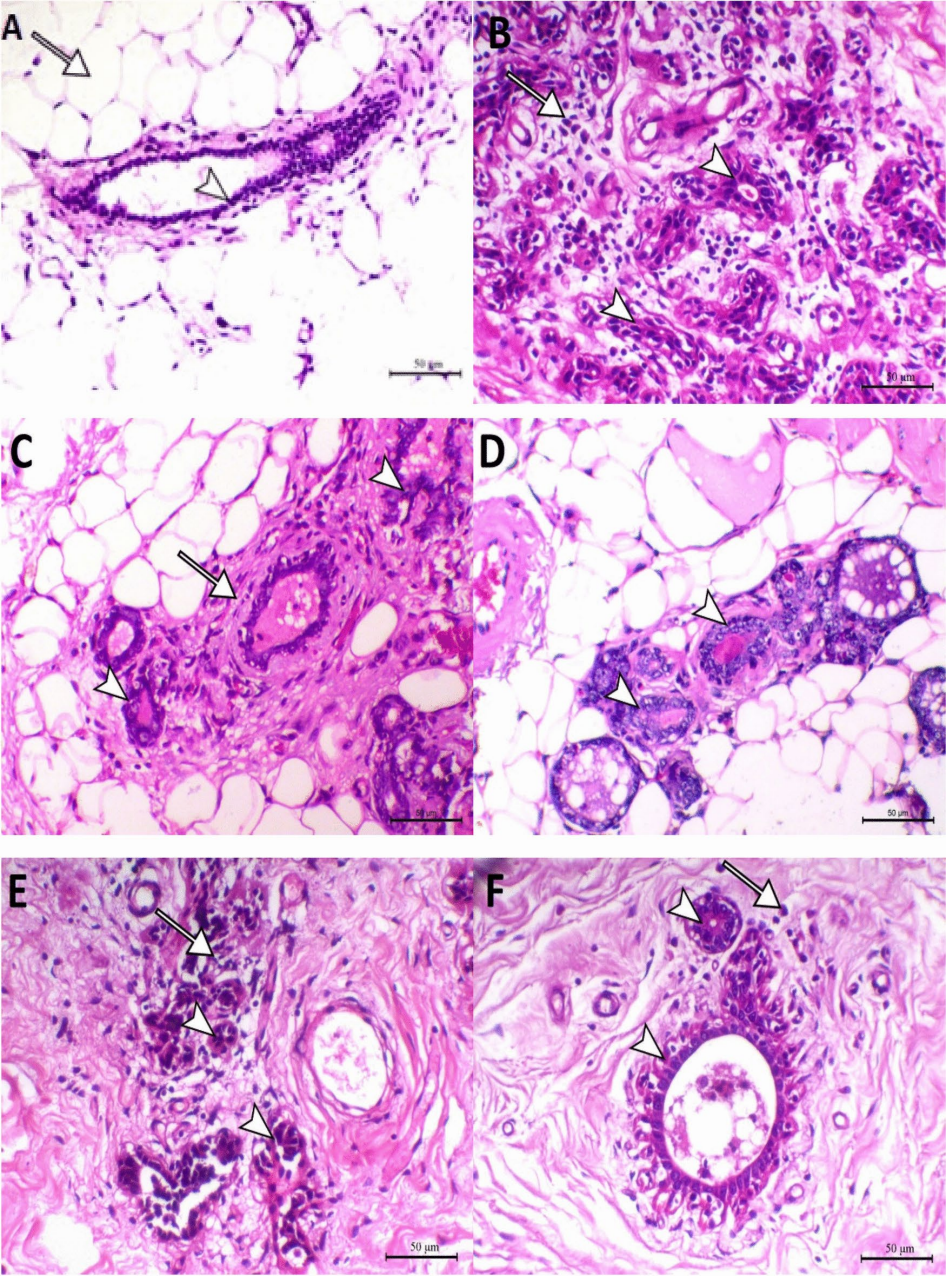
Mammary gland cross-sections photographed under a microscope for the different groups are shown in slide A as the control group, B slide as the DMBA carcinogenic group, C slide as the amygdalin treatment group, D slide as amygdalin protective group, E slide as the tamoxifen therapy group, F group as amygdalin combined with tamoxifen therapy. X&E, X200, bar = 50 µm.

The histopathological analysis in Fig. 5 shows that the negative control group has a glandular duct lined with epithelial lining (arrowhead – Fig. 5. A) within a fat stroma (arrow indicates fat cell—Fig. 5. A). Photomicrographs of the cross-section of the carcinogenic mammary gland are shown in Fig. 5. B has proven the success of the current method, materials, and duration used in this in vivo experiment to induce mammary tumors in mice. DMBA group had proliferated microductular carcinoma (arrowheads – Fig. 5. B) associated with myxomatous changes within the connective tissues matrix, prominent angiogenesis, and marked mononuclear inflammatory cell infiltration (arrowhead indicates plasma cells). However, the mammary gland of DMBA mice treated with amygdalin showed marked decreases in the proliferated ducts (arrowheads – Fig. 5. C) with a significant increase of extracellular matrix around the duct (arrow – Fig. 5. C). Furthermore, the mammary gland of the protected group showed hyperplastic glandular acini with marked tubular basophilia (arrowheads – Fig. 5.D).

On the other hand, the mammary gland of DMBA treated with tamoxifen showed marked degenerated and apoptotic changes within the glandular acinar epithelium (arrowheads – Fig. 5. E) and moderate periglandular inflammatory cells infiltration (arrow – Fig. 5. E). Thus, the mammary gland of DMBA mice with a combination of both tamoxifen and amygdalin showed a marked decrease in the increased ducts (arrowheads – Fig. 5. F), an increase in periglandular myoepithelial cells, and a substantial reduction in the inflammatory cells infiltration (arrow indicates few plasma cells – Fig. 5. F).

## Discussion

The research results proved that amygdalin possesses a Protective and Chemotherapeutical Role in Induced Mammary Cancer in Experimental Mice and Upregulation of Related Genes, wherein it has excellent efficacy as used alone as a treatment or a protective drug or in co-administration with tamoxifen therapy in DMBA-treated mice in liver enzymes, tamoxifen therapy reached the hepatic values to be within the control range, more preferable to Amygdalin treatment than Amygdalin co-administration with tamoxifen therapy over Amygdalin protection, respectively. As the serum activity of the liver cytoplasmic enzyme increases in carcinogenic mice, ALT indicates necrotic lesions in the hepatic cells^49^. Thus, Amygdalin administrations have hepatic ameliorative potential against DMBA carcinogen, with increases in albumin and decrease in liver enzyme activity (alanine transaminase (ALT), aspartate transaminase (AST), as well as alkaline phosphatase (ALP)) compared to carcinogenic mice^50^. Amygdalin significantly decreased serum hepatic function, while DMBA-carcinogenic group serum AST, ALT, and ALP increased. This protects the liver by maintaining plasma membrane integrity and inhibiting enzyme leakage. Because of this, the extract’s ability to restore the activities of marker enzymes following delivery may be explained^51^.

Regarding kidney functions, the results showed that Amygdalin protection proved more potential than Amygdalin co-administration with tamoxifen therapy and Amygdalin treatment than tamoxifen therapy, respectively. Reductions in renal function concentrations demonstrated that amygdalin has a renal ameliorative effect against renal damage in female mice. Our findings corroborated those of Guo et al.^52^, who found that amygdalin prevents the rise in blood urea and serum creatinine in cases with chronic renal disease. On the other hand, increased renal function levels in the blood are another side effect of DMBA cancer due to Extracellular matrix (ECM) buildup and fibroblast proliferation in the kidney^18,53^. Moreover, the inhibition of kidney cell cancer development by amygdalin was reported by Juengel et al.^54^, and this effect was reversed after treatment with amygdalin.

DMBA-induced breast cancer had higher estrogen and prolactin levels than controls. According to statistical research, amygdalin protection decreased estrogen steroid hormone (E2) more than co-administration with tamoxifen therapy or treatment alone. Prolactin (PRL) steroid hormone decreased more with amygdalin co-administration with tamoxifen therapy than with tamoxifen alone, Amygdalin protection, or Amygdalin treatment in DMBA mice. Amygdalin oral administration significantly reduced endocrine estrogen and anterior pituitary prolactin levels in DMBA group mice^55^. This means that the hypothalamic-pituitary-ovarian axis, its hormones, growth factors, receptors, and oxidative balance are all involved in carcinogenesis and female reproduction processes via regulating mammary cell survival and secretion by Amygdalin^56^. Tamoxifen therapy has mixed estrogenic and antiestrogenic effects. Therefore, considerable improvements in E2 and PRL hormones were noted^5^.

Other aspects revealed a significant decrease in tumor markers of carcinoembryonic antigen (CEA), cancer antigen 15.3 (CA15.3), and cancer antigen 125 (CA125) with Amygdalin administrations, whether in treatment or protection group inconsistent with Jaswal et al.^57^. In antibody-directed enzyme prodrug treatment, tumor-specific antibodies were conjugated to enzymes and then given systemically to combat tumor antigens (ADEPT). The enzyme then helped the prodrug become an effective cytotoxic agent that could be used directly on the tumor. The duration between dosages was optimized to reduce systemic toxicity by clearing the tumor’s blood and normal tissues of the conjugates. As the functional medicine travels to the malignancy, it will cause the bystander effect and kill the antigen-negative cells^58^. The tumor has been treated using amygdalin, a prodrug that may be broken into free cyanide by the sweet enzyme almond α-glucosidase. If activated locally at the tumor site, this chemical could eliminate malignant tumor cells without causing systemic damage. Some believed this drug might kill cancer cells by attaching to the antibody. In conjunction with the ADEPT system, amygdalin and beta-glucosidase were considered promising targeted cancer therapy^59^.

From current results of tumor markers, tamoxifen proved to be more highly effective than amygdalin, but amygdalin in co-administration with tamoxifen therapy showed excellent efficacy on breast tumor markers. According to estrogen antagonism of tamoxifen therapy, the mammary tumor marker CA15.3 and general tumor marker CEA significantly decreased in carcinogenic mice, but CA125 significantly decreased in carcinogenic mice due to its potentiality on inhibition of progesterone receptors in the endometrium^60,61^.

The effect of Amygdalin protection on malignant breast tissue GPx was much greater than that of treatment, co-administration with tamoxifen, or tamoxifen alone. The current study showed that amygdalin effectively controlled the antioxidant defense system throughout the experiment by regulating superoxide dismutase (SOD) and glutathione peroxidase (GPx) activities, which are indicative of amygdalin’s antioxidant properties and free radical scavenging capacity. Similarly, Karabulut et al.^62^ demonstrated that Amygdalin supplementation offered robust protection against the oxidative stress generated by DMBA by lowering oxidative stress and raising both SOD and GPx values. In a mouse model of 7,12-dimethylbenz[a]anthracene (DMBA)-induced carcinogenesis, the Amygdalin-containing fraction showed anti-cancer efficacy in vivo by boosting the antioxidant response as evaluated by SOD and GPx activities. Amygdalin was found to play a role in the functioning of the Amygdalin-containing fraction. The latter could be metabolized into HCN in malignant tissue, leading to cell death via oxidative stress^63^.

Furthermore, compared to the DMBA carcinogenic group, after 4 weeks, the therapeutic dose of tamoxifen increased the activity of SOD and GPx values. The findings of Lim et al.^64^, who found that tamoxifen inhibits hydrogen peroxide production, corroborated these findings. Tamoxifen’s antioxidative potential can be explained as follows. Tamoxifen is metabolized into 4-hydroxy tamoxifen and N-desmethyl tamoxifen by the liver’s cytochrome P450, a dependent oxidase, during tamoxifen mediation. This antioxidant property of breaking chains and the possible impacts on membrane structure result from the phenolic hydroxyl group^65^.

Tumor necrosis factor-alpha (TNF-α), a potent paracrine and endocrine modulator of inflammatory and immunological processes, played a significant role in evaluating the anti-cancer effect of amygdalin in this study through gene expression. B lymphoma cell 2 (BcL-2) which is an anti-apoptotic marker; caspase-3, which is an apoptotic marker; and 53 K. Dalton Protein (P53), which acts as a tumor suppressor and regulates cell division by keeping cells from growing and dividing (proliferating) too fast or in an uncontrolled way^66^.

Compared to the DMBA group, Amygdalin protection dramatically reduced malignant breast tissue TNF-α more than tamoxifen therapy, co-administration, or treatment alone. The effect of Amygdalin co-administration with tamoxifen therapy on malignant breast tissue BcL-2 was significantly more down-regulating than treatment, protection, or DMBA. Additionally, Amygdalin co-administration with tamoxifen therapy upregulated malignant breast tissue caspase-3 more than tamoxifen therapy, Amygdalin treatment, or Amygdalin protection compared to the DMBA group. The apoptosis cycle was promoted by two pathways: (1) the death receptor-mediated extrinsic apoptosis pathway and (2) the intrinsic mitochondrial pathway. Apoptosis family proteins, such as Bcl-2, work by neutralizing pro-apoptotic proteins like Bax, which protects the tumor cells from apoptosis by occurring in the cytosol and then translocating to the mitochondria to induce apoptosis^67,68^. The mitochondrial system, which encompasses both pro- and anti-apoptotic proteins, is regulated by Bcl-2^67^.

Compared to the DMBA group, Amygdalin treatment upregulated malignant breast tissue P53 more than tamoxifen therapy, co-administration with tamoxifen therapy, or prevention. Both TNF-α and BcL-2 gene expressions were significantly increased by DMBA action, but both caspase-3 and P53 gene expressions were significantly decreased. The mechanisms of transcriptional inhibition that reflect the ability of androgen receptors to bind DNA included two models. Type 1 antagonists bind the receptor but hinder receptor DNA binding, whereas type 2 antagonists induce DNA binding but fail to initiate gene transcription^69^.

In contrast to B cell acute lymphoblastic leukemia, which is positive in roughly half of the cases, and neoplastic plasma cells of multiple myeloma, which are CD20-negative, our flow cytometric research demonstrates that over 90% of cases with non-lymphomas Hodgkin’s are CD20-positive^70^; and CD44 which is an early indicator for induction of breast cancer and act as an immune suppression in late breast carcinoma^71^.

According to CD20 and CD44 data, Amygdalin co-administration with tamoxifen therapy affects malignant breast tissue. Compared to the DMBA group, Amygdalin treatment, tamoxifen therapy, and Amygdalin protection downregulated CD20 and CD44 considerably. In agreement with the reports mentioned above, the current study demonstrated over-expression of CD20 and CD44 protein in DMBA-induced mice compared to control mice^72^. In breast cancer cases, the epithelial-mesenchymal transition state is linked to a cancer stem cell-like population harboring the CD44 + /CD24- profile and has been proposed to play a critical role in metastatic progression^73^. As mentioned in our discussion and correlation with other literature, up-regulation of CD20 and CD44 has been a marker for breast cancer^74^.

Carcinogenesis is linked to cell cycle checkpoint gene loss and apoptosis suppression. Cell cycle checkpoint genes regulate DNA replication and chromosomal segregation to ensure cell cycle progression. Normal cells’ checkpoint genes temporarily block the cell cycle by increasing gene transcription after DNA damage. This aids DNA repair to prevent mitosis-transmitted DNA lesions. Incomplete DNA repair stops the cell cycle and activates apoptosis. DNA instability caused by checkpoint gene loss can turn normal cells into cancer cells^75^. Thus, cell cycle analysis is necessary to investigate tumor cell proliferation and inhibition.

The effect of Amygdalin co-administration with tamoxifen therapy on malignant breast tissue in the sub-G1 phase was substantially more significant than that of Amygdalin treatment, tamoxifen therapy, or protection compared to the DMBA group. Compared to the DMBA group, tamoxifen therapy had a considerably more significant effect on malignant breast tissue in the G0/1 phase than Amygdalin treatment, protection, or co-administration. Instead, compared to the DMBA group, Amygdalin protection on malignant breast tissue in the S. phase and G2/M was substantially greater than Amygdalin treatment, tamoxifen medication, or Amygdalin co-administration with tamoxifen therapy. This study found typical cell cycle phases in control mice’s mammary tissues. Mammary tumor induction caused uncontrolled cell invasion at various periods. Our findings support previous findings that cell cycle disruption contributes to tumor growth^76^. Furthermore, we find the disturbances in cell cycle machinery, i.e., the proportional increase in sub-G1 or G0/1 phases and decrease in synthesis (S) and Gap2/Mitotic (G2/M) phases at acute and protracted treatments^77^. Thus, plant-derived compounds have been reported to induce cell cycle arrest and cell death in many tumor cell lines^78^. The prophylactic and therapeutic effects of Amygdalin shift of cell distribution into the G0/1 phase. Meanwhile, the proportion of cells in the S and G2/M phases was significantly decreased compared to the tumor-bearing untreated group (DMBA group).

According to an apoptotic marker (Annexin V), tamoxifen therapy increased malignant breast tissue in the UL region more than the Amygdalin treatment, co-administration, or protection compared to the DMBA group. Tamoxifen therapy increased the effect of malignant breast tissue in the LL region more than Amygdalin treatment, protection, or administration with tamoxifen therapy compared to the DMBA group. Compared to the DMBA group, Amygdalin co-administration with tamoxifen medication increased malignant breast tissue in the UR region more than Amygdalin treatment, protection, or isolation. Compared to the DMBA group, Amygdalin protection on malignant breast tissue in the LR region was substantially greater than amygdalin treatment, tamoxifen therapy, or Amygdalin co-administration with tamoxifen. In this respect, such a strategy perhaps partially agrees with the cytotoxic mechanisms of action of the tested compound^79^. Early apoptosis implies positive for annexin and negative of propidium iodide, while late apoptosis and necrosis are positive for both annexin and propidium iodide.

In histopathology, our study showed that histopathological findings agreed with our molecular and biochemical results, showing the modulatory effect of different treatments on mammary gland cancer cells. Histopathological findings of DMBA-induced mice showed neoplastic activity detected in the acini and ductal epithelium, leading to cystic adenoma. Still, the mammary gland of DMBA mice treated with amygdalin registered a massive recurrence of normal histological structure appearance of a few acini tissue sections. In the amygdalin-protected group, the high-grade mammary intraepithelial neoplasia histological pattern was replaced by typical mammalian acini in the mammary gland. These outcomes might occur from the decomposition of amygdalin into benzaldehyde, glucose, and hydrocyanic acid^17^.

On the other hand, the mammary gland of DMBA treated with tamoxifen depleted the periglandular inflammatory cells infiltration. In contrast, the mammary gland of DMBA mice with a combination of both tamoxifen and amygdalin showed a marked decrease in the proliferated ducts, an increase of periglandular myoepithelial cells, and a marked reduction of inflammatory cells infiltration. These findings were related to the role of tamoxifen as a partial agonist of the estrogen receptors^5^, rather than amygdalin decomposition.

## Conclusions

Breast cancer in female mice induced by the DMBA carcinogen may be treated with amygdalin, delivered either separately (as a prophylactic or therapeutic drug) or in combination with tamoxifen therapy. The biochemical markers of liver function (AST, ALT, and ALP) and renal function (urea, creatinine), raised due to DMBA exposure, returned to baseline levels after amygdalin treatment. Amygdalin improves breast cancer by reducing the levels of steroid hormones (E2 and PRL) and blood tumor markers (CEA, CA15.3, and CA125). Amygdalin demonstrated considerable antioxidant activity by enhancing the activities of SOD and GPx in breast cancer tissue. Conversely, the gene expressions of caspase-3 and P53 were elevated, whereas TNF-α and BcL-2 were downregulated in breast tissue. Flow cytometric research indicated that amygdalin was associated with CD20 and CD44, facilitating the cell cycle and death in carcinogenic mice. Histopathological tests supported all the aforementioned data, revealing that the DMBA group exhibited proliferative microductular carcinoma accompanied by significant mononuclear inflammatory cell infiltration, which diminished following the amygdalin administration. Amygdalin, a new anti-cancer drug, significantly protected breast tissue from experimentally induced apoptosis in female mice with mammary carcinogen-induced tumors. It is advisable to integrate various modalities of pharmaceutical combinations to effectively administer amygdalin along the multiple pathways implicated in the development and progression of breast cancer.

## Data Availability

Data is provided within the manuscript.
